# Ultrasound-Assisted Esterification of Valeric Acid to Alkyl Valerates Promoted by Biosilicified Lipases

**DOI:** 10.3389/fchem.2018.00197

**Published:** 2018-06-07

**Authors:** Soledad Cebrián-García, Alina M. Balu, Rafael Luque

**Affiliations:** ^1^Departamento de Quimica Organica, Universidad de Cordoba, Cordoba, Spain; ^2^Scientific Center for Molecular Design and Synthesis of Innovative Compounds for the Medical Industry, Peoples Friendship University of Russia (RUDN), Moscow, Russia

**Keywords:** biosilicification, esterification, ultrasounds, biocatalysis, lipases

## Abstract

A novel, environmentally friendly, and sustainable ultrasound-assisted methodology in the valorization of valeric acid to alkyl valerate using a biosilicified lipase from *Candida antarctica* is reported. This one-pot room temperature methodology of enzyme biosilicification leads to biosilicified lipases with improved activity and reaction efficiency as compared to free enzymes. Yields in the ultrasound-promoted esterification of valeric acid was ca. 90% in 2 h with 15% m/v of biosilicified lipase (Bio-lipase; 616 U/g _biocatalyst_ enzymatic activity) and a molar ratio 1:2 (valeric acid:ethanol), slightly superior to that observed by the free enzyme (75% conversion, 583U/g _biocatalyst_ enzymatic activity). The reuse of enzymes in these conditions was tested and the results show a relatively good reusability of these biosilicified enzymes under the investigated conditions, particularly preserving fairly stable specific activities (616 vs. 430 U/g _biocatalyst_ after four reuses).

## Introduction

The valorization of waste generated in various industrial activities worldwide becomes fundamental for the development of a sustainable planet and a more sustainable society. Esters of carboxylic acids are generally recognized natural flavors and fragrances with numerous applications (Dhake et al., 2013). Ethyl valerate, with a typical green apple fragrance, arouses great interest since it is widely used in the food, cosmetic, and pharmaceutical industries (Karra Chaabouni et al., 2006; Padilha et al., 2013). Importantly, alkyl valerates have also been recently demonstrated to have excelling properties to be employed in biofuel blends (Hari Krishna et al., 2001). These components, commonly extracted from natural resources, are however very limited in nature (Hari Krishna et al., 2001; Ben Akacha, 2014). Additionally, the chemical synthesis of esters generally employ not particularly green methods (harsh reaction conditions, use of mineral acids, etc.), with most systems exhibiting low selectivity (Ben Akacha, 2014; Brault et al., 2014). Lipases (triacylglycerol ester hydrolases—EC 3.1.1) are a promising alternative in esterification reactions, particularly for valeric acid, under moderate conditions (Adlercreutz, 2013; Dhake et al., 2013; Ben Akacha, 2014). Furthermore, the production of these esters via enzymatic route have been considered as more environmentally friendly (Brault et al., 2014; Corradini et al., 2017).

Biocatalytic processes, mediated by enzymes, have attracted keen interest in the valorization of biomass (Himmel et al., 2007). They represent a more efficient and ecological alternative to the traditional inorganic catalysts due to the fact that they take place under environmentally friendly conditions (room temperature, neutral pH), with often greater yields because of their regioselectivity and stereoselectivity (Patel et al., 2017). On the other hand, the use of these biocatalysts is not well established in industry because they have two main disadvantages: their limited stability, not compatible with continuous production (Itabaiana et al., 2013); and the difficulty of separating the substrates and products in the reaction medium, which prevents their reuse (Corradini et al., 2017). The immobilization of enzymes was proposed to partially overcome these problems, and allowed to date a number of biocatalytic industrial processes to be economically profitable (Güven et al., 2002).

A proposed enzymatic immobilization implemented by our group is the use of silica (SiO_2_) as encapsulating material, the proposed process denoted biosilicification. Enzyme biosilicification is based on a simple sol-gel synthetic procedure in which a SiO_2_ precursor (e.g., tetraethylorthosilicate) is added dropwise to a buffer solution of the commercial enzyme to generate a partially encapsulated enzyme in a SiO_2_ material upon hydrolysis of the SiO_2_ precursor (Itabaiana et al., 2013). The biosilicification was proved to give the enzyme a greater stability (at room temperature and neutral pH) in organic and aqueous media and to increase the catalytic efficiency at the same time. This is due to an observed partial encapsulation of the enzyme by the porous SiO_2_ that provides a mechanical/thermal stability to the enzyme. Additionally, it favors recovery and reuse, in comparison to the free enzyme, reducing the final costs of production (Pistone et al., 2016).

One of the novel methodologies that attract significant attention in biocatalyzed esterification reactions is the use of ultrasonic systems, alternative greener processes with high yields, and a greater selectivity as compared to other methodologies (Adak and Banerjee, 2016; Gupta S. et al., 2017; Gupta S. M. et al., 2017; Javad Safaei Ghomi, 2018). By comparison with conventional heating in carousel reactors, where mass transfer in esterification reactions is very limited, acoustic cavitation, and ultrasound hydrodynamics can solve mass transfer limitations at short times of reaction (Singh et al., 2007; Mahamuni and Adewuyi, 2009; Thanh le et al., 2010; Batistella et al., 2012; Veljković et al., 2012; Bhangu et al., 2017). This methodology also increases the yield in the reaction mediated by biocatalyst under the pressure and temperature conditions required for lipases (Suslick, 1990; Eddingsaas and Suslick, 2006). In summary, this methodology shows low reaction times, does not require the use of toxic solvents (Xu et al., 2013), being low energy intensive and cost-competitive.

Following previous efforts from the group in the design of biosilicified enzymes (Itabaiana et al., 2013; Henriquez et al., 2014; Pistone et al., 2016) the objective of this study was to evaluate the performance and stability of biosilicified lipases in the esterification of valeric acid under ultrasonic conditions. Parameters including type of alcohol, acid: alcohol ratio and temperature were investigated. The immobilized lipase was characterized using different BET, TGA, and XPS techniques and subsequently tested for stability and reuses under the investigated ultrasound-assisted conditions.

## Results

### Esterification activity

**Scheme 1:**
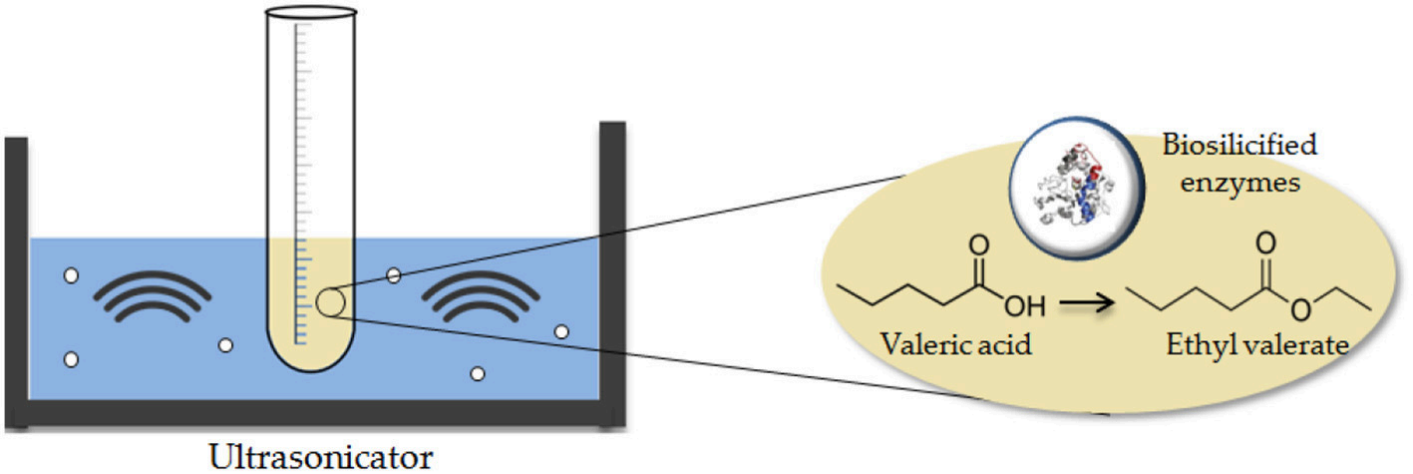
Ultrasound-assisted esterification of valeric acid catalyzed by biosilicified lipases.

Esterification experiments (Scheme 1) were conducted at room temperature via ultrasounds at different acid: alcohol molar relations and diverse concentrations of biocatalyst.

Table 1 summarizes the main set of experiments under optimized reaction conditions. Blank runs (in the absence of biocatalyst) or in the presence of a SiO_2_ material (without enzyme synthesized using an identical methodology in the absence of enzyme buffer solution) did not provide any valeric acid conversion under the investigated ultrasonic-promoted conditions despite the use of different alcohols and acid:alcohol ratios (Table 1, entries 1–8).

**Table 1:** Ultrasound-assisted esterification of valeric acid with various alcohols (MeOH, EtOH, iPrOH, BuOH) and different acid: alcohol ratios.

**Entry**	**System**	**Alcohol**	**Acid:alcohol Ratio**	**Conversion (mol%)**	**Specific activity (U/g _biocatalyst_)**
1	Blank	Methanol	All	–	–
2		Ethanol			
3		1-Propanol			
4		1-Butanol			
5	SiO_2_ material	Methanol	All	-	–
6		Ethanol			
7		1-Propanol			
8		1-Butanol			
9	Free enzyme	Ethanol	1:1	75	583 ± 16
10			1:2	76	
11			1:3	74	
12	Bio-lipase	Methanol	All	–	–
13					
14					
15		Ethanol	1:1	85	616 ± 9
16			1:2	90	
			1:3	82	
17					
18		1-Propanol	1:1	50	480 ± 6
19			1:2	60	
20			1:3	57	
21		1-Butanol	1:1	73	585 ± 14
22			1:2	76	
23			1:3	75	

Reaction conditions: 0.01 mol valeric acid (1.09 mL), 0.01–0.03 mol EtOH (MeOH, i-PrOH, BuOH), 30 mg Bio-lipase (15% m/v, equal quantity for free enzyme in buffer solution—corresponding to 83.3 μL—), 2 h ultrasonic irradiation. Specific activities calculated for 1:2 acid:alcohol ratio (selected optimum conditions).

The enzymatic activity measured in all experiments (Table 1) provided comparable specific activities for the free enzyme (even slightly reduced, Table 1, entry 16) to Bio-lipase. Taking into consideration the quantity of free enzyme in the systems, the calculated percentage of protein loading in biosilicified lipase was 86%. These results are in good agreement with previous reports which related this comparable/improved activities at slightly reduced protein loadings to the stability of the encapsulated/immobilized lipase (Itabaiana et al., 2013; Pistone et al., 2016). Comparably, the highest conversion (90% conversion, 100% selectivity to ethyl valerate) was obtained using a 15% m/v of biosilicified enzyme and a 1:2 acid: alcohol molar ratio after 2 h (Table 2, entry 16).

**Table 2:** Effect of the quantity of biosilicified lipase and water content in the **c**onversion of valeric acid to ethyl valerate.

**% Biosilicified lipase (m/v)**	**Conversion ethyl valerate (%)**	**Water (μL)**	**Conversion ethyl valerate (%)**
15	82	400	69
7.5	65	300	55
5	38	200	54
2.5	<20	100	58

*Reaction conditions, 0.01 mol valeric acid (1.09 mL), 0.02 mol EtOH (1.16 mL), 2 h ultrasonic irradiation*.

Additional investigations on different acid:alcohol ratios (Table 1, entries 15–17, 18–20, and 21–23) indicated that the acid-to-alcohol ratio did not have a significant influence in the specific activities and/or in the conversion in the systems under the investigated reaction conditions. In any case, a 1:2 acid:alcohol ratio was selected as optimum in all experiments. Interestingly, the use of other alcohols (e.g., 1-Propanol and 1-Butanol) could provide conversions to alkyl valerate between 50 and 70% (Table 1, entries 18–23), with minor differences also between more acid/alcohol concentrated solutions.

Conversions in the 70–82% range at complete selectivity could be also obtained keeping the quantity of enzyme to a maximum of 15% m/v and/or in aqueous solution (400 μL water added, Table 2). The presence of water in the reaction has been reported to favor enzyme-substrate interaction in biocatalyzed reactions (Bradford, 1976; Garcia-Galan et al., 2014), which in this case was not significantly influenced for biosilicified lipases under the investigated reaction conditions (Table 2, water results). A decrease in catalyst loading was found to decrease, as expected, ethyl valerate yields and specific activities of Bio-lipase and the free enzyme in the investigated reactions (Table 2, %biosilicified lipase results).

A probe was employed in the ultrasound system for temperature control and to determine the increase in temperature in the medium during the first hour of reaction. After this time period, cool water replaced the original solution to start a new reaction time. Over the hour reaction period, the temperature increases by ca. 20°C (max. 40–45°C), with such observed temperature increase being reproducible and consistent per hour for several cycles.

The stability and reusability of the enzyme under the investigated ultrasonic irradiation conditions was subsequently tested (Table 3). Results pointed out that the biosilicified lipase gradually deactivates with reuses in ethanol (from 90 to 60% after four uses) but importantly Bio-lipase was not fully denaturalised after several cycles under the investigated conditions. Reused biosilicified enzymes, as expected, provided after equal ultrasound treatment reduced specific activities (300-400 U/mg biocat., reuses R2-R4) as well as lower protein loading ratios (70-53% protein content in reused R_2_, R_3_, and R_4_ biosilicified lipase vs. 86% protein present in the fresh Bio-lipase) related to the loss of protein with reuses which seems to account for deactivation. Indeed, additional experiments on the potential activity/conversion of leached enzyme (reduced protein content after reuses, Table 3) indicate that such enzyme loss after reuses has exclusively residual activity (21–15% conversion, almost negligible specific activity) indicating (1) enzyme loss after reuses does not account for any relevant activity in the systems; (2) while it does certainly account for the observed deactivation after each cycle (Table 3, conversion values).

**Table 3:** Reusability studies of biosilicified lipase in the esterification reactions using ultrasound.

**Conversion alkyl valerate (%)**				**Specific activity**	**Protein loading**
	**Alcohols**			**(U/g _biocatalyst_)**	**(%)**
**Reuses**	**Ethanol**	**1-Propanol**	**1-Butanol**		
R_0_	90 ± 4	43 ± 6	72 ± 3	616 ± 9	86
R_1_	84 ± 8	45 ± 4	70 ± 2	586 ± 7	82
R_2_	78 ± 3	40 ± 6	76 ± 3	524 ± 11	70
R_3_	68 ± 6	46 ± 2	60 ± 6	465 ± 8	54
R_4_	60 ± 2	42 ± 1	54 ± 4	430 ± 9	53

*Reaction conditions, 0.01 mol valeric acid (1.09 mL), 0.02 mol EtOH/ i-PrOH/ BuOH (1.16 mL, 1.53 mL, 1.83 mL, respectively), 30 mg biosilicified lipase (15% m/v), 2 h ultrasonic irradiation*.

Interestingly, Bio-lipase was found to be increasingly stable in 1-Propanol and 1-Butanol. Selectivity to alkyl valerate was fully preserved after reuses in each case. Comparably, the free enzyme provided a 72% conversion at complete selectivity in the first run, followed by a significant reduction in specific activity after 2 reuses (<20% conversion, <150 U/mg biocat., run R_2_), most probably due to denaturalisation under ultrasonic conditions.

### Characterization of the biosilicified enzyme materials

**Figure 1:**
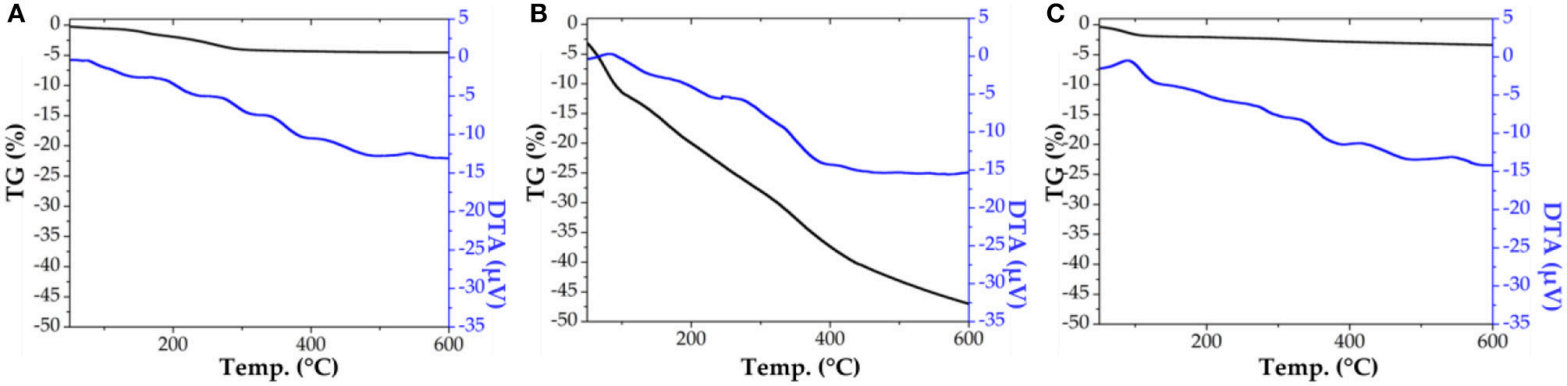
Thermogravimetric (TG) analysis for SiO_2_ support **(A)**, biosilicified lipase R_0_ **(B)**, and biosilicified lipase R_3_ **(C)**.

The characterization of SiO_2_ material (Bio-lipase, same protocol in the absence of the enzyme buffer solution) showed a very similar surface area, exhibiting characteristics of non-porous materials prior to template removal [pores blocked with n-dodecylamine template, see also Figure 1 for Thermogravimetric (TG) analysis results]. Upon template removal (soxhlet extraction in ethanol under reflux for 8 h), the SiO_2_ material exhibited large surface areas (ca. 500 m^2^ g^−1^), with a pore diameter of 3.6 nm, typical of organo-templated mesoporous SiO_2_ materials (Macquarrie et al., 2005).

Comparably, the textural properties of the fresh as-synthesized biosilicified enzyme (R_0_) show BET surface values below 5 m^2^/g^−1^, with negligible pore volumes (Table 4). These results indicate that the biosilicifed enzyme is essentially non-porous. However, these new biological catalysts do not have to meet the typical surface-specific standards normally common in inorganic solid catalysts. These materials may not have a direct relationship between catalytic surface/biocatalyst activity as illustrated in the biocatalytic activity results. Interestingly, thermogravimetrically treated Bio-lipase materials exhibited an interesting behavior worth discussing. The total loss of organics from biosilicified lipase R_0_ after TG (Figure 1, middle graph, 47.5% weight loss in the 110–600°C range) rendered a nicely porous material that still contains certain quantities of lipase (ca. 35–45% protein loading) and remains active in the investigated esterification reactions. Comparably, the third reused Bio-lipase (R3) material after thermogravimetric treatment (heating up to 600°C) exhibited a largely similar surface area (443 m^2^/g^−1^) as well as textural properties (pore volume and diameter) for which a minimum mass loss was observed in the 100–600°C range (ca. 4% from its original weight), accounting for ca. 45–50% protein loading left in the material. Taking into account the measurements errors and observed similarities, these relevant results further support the high stability of the enzyme in the biosilicified form as compared to the free enzyme, particularly under the investigated ultrasonic irradiation conditions.

**Table 4:** BET surface area and pore volumes of biosilicified enzymes (as-synthesized and after TG treatment) as compared to analogously prepared silica materials (without enzyme).

**Material**	**S_BET_^a^(m^2^g^−1^)**	**V_BJH_^c^(cm^3^g^−1^)**	**D_BJH_^b^ (nm)**
SiO_2_ material	<10	–	–
SiO_2_ template removed (soxhlet)	502	0.8	3.6
As-synthesized Bio-lipase (R_0_)	<5	–	–
Bio- lipase (R_0_)-after TG heating-	322	0.2	4.5
Bio- lipase (R_3_)-after TG heating-	443	0.4	4.8

a *S_BET_, specific surface area was calculated by the Brunauer–Emmet–Teller (BET) equation*.

b *DBJH, mean pore size diameter was calculated by the Barret–Joyner–Halenda (BJH) equation*.

c *VBJH, pore volumes were calculated by the Barret–Joyner–Halenda (BJH) equation*.

**Figure 2:**
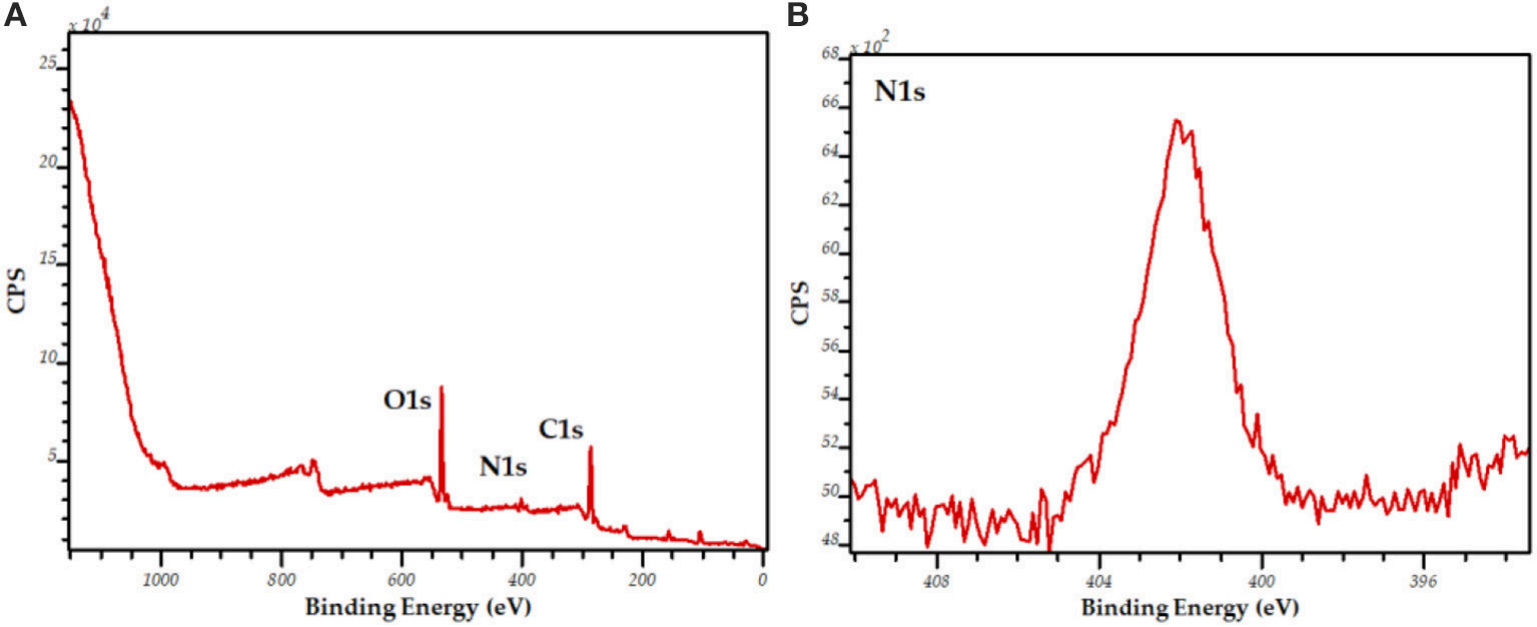
XPS spectra [survey, **(A)**; N1s, **(B)**] of biosilicified lipase.

The broad and intense XPS bands of N and C1s demonstrate the presence of C and Ni on the surface of the biosilicified material (Figure 2). These results may indicate a potential availability of the enzyme on the surface of the biosilicified material in spite of the biosilification process which rendered highly active encapsulated enzymes.

## Conclusions

A simple and efficient methodology is reported for the highly selective esterification of valeric acid to alkyl valerate using synthesized encapsulated lipases (via biosilicification) under ultrasonic irradiation. Biosilicified enzymes exhibited slightly improved specific activities and product yields in the systems as compared to the free enzyme in ethanol (90% conversion and 616 U/g _biocatalyst_ vs. 75% conversion and 583 U/g _biocatalyst_ at complete ethyl valerate selectivity after 2 h reaction under ultrasounds). Importantly, biosilicified lipase materials were active in the esterification using longer chain alcohols (e.g., 1-Propanol, 1-Butanol) also exhibiting a moderate to good reusability after several uses, with a stable specific activity despite the significant protein loss (616 vs. 430 U/g _biocatalyst_ with a reduction in protein content from 86 to 53% after four reuses). The observed protein loss proved to be responsible for the slight deactivation observed after reuses but clearly had a minor contribution in the activity of the systems. Comparatively, biosilicified lipases were remarkably more stable than the free enzyme that fully deactivates after two reaction runs.

## Materials and methods

### Materials

All reagents were purchased in Sigma Aldrich and were used without further purification. The enzyme lipase B of the species *Candida antartica* (CalB) was purchased from Sigma Aldrich as a buffer solution.

### Enzyme biosilicification

According to the protocol of Henriquez et al. (2014), Tetraethoxysilane (TEOS) (20.80 g, 0.10 mol) is added to a stirred solution composed of n-dodecylamine (5.10 g, 0.03 mol), 50 g acetonitrile, and 50 g buffer solution of Cal-B at room temperature. The solution containing a visible solid precipitate after a few minutes and was stirred for another 3 h, then filtered, washed with ethanol, and dried at room temperature for at least 24 h. The SiO_2_ material was synthesized using the same protocol without the addition of the buffer solution of Cal-B.

### Protein concentration and enzymatic activity

Protein concentration was defined by Bradford method (Bradford, 1976), using as standard protein bovine serum albumin. All solutions were prepared by employing ultrapure water.

Enzymatic activity was determined by hydrolysis of p-nitrophenyl butyrate (pNPB), in acetonitrile, pH 7 and 25°C according to the protocol described by Garcia-Galan et al. (2014). P-nitrophenol, released to the reaction medium as hydrolysis product of pNPB, was measured at 400 nm. In the present work, a unit (U) is the amount of enzyme capable of hydrolyzing 1 μmol of substrate per minute at pH 7 and 25°C.

### Immobilization efficiency

Immobilization efficiency was evaluated with two parameters: Protein loading ratio and activity yield. Protein loading ratio (%) was used to compare the amount of enzyme that finally remains into the biocatalyst with respect to the free enzyme (for which a value of 100% is given). Activity yield (%) equally determined the difference between specific activity in case of biosilicified enzyme and free form.

(1) $$\begin{array}{l} \text{Protein loading ratio }\left(\%\right)=\frac{\text{protein loaded}}{\text{protein introduced}}\text{ x }100\;\% \end{array}$$

(2) $$\begin{array}{l} \text{Activity yield }\left(\%\right)=\frac{\text{specific activity }\left(\text{immobilized lipase}\right)}{\text{specific activity }\left(\text{free lipase}\right)}\text{ x }100\;\% \end{array}$$

### Materials characterization

The immobilized enzymes were characterized using N_2_ physisorption and powder X-ray diffraction (XRD). N2 adsorption measurements were performed at 77 K by using a Micromeritics ASAP 2000 volumetric adsorption analyzer. The samples were degassed at 35°C for 24 under vacuum (*p* < 10¢2 Pa) before the adsorption measurements. Surface areas were calculated according to the BET (Brunauer–Emmet–Teller) equation. Mean pore size diameter and pore volumes were measured from porosimetry data by using the BJH (Barret–Joyner–Halenda) method.

TGA measurements were performed using a System Setaram Setsys 12 TGA instrument. Samples were heated at a heating rate of 10°C min^−1^, in Nitrogen (50 mL min^−1^) at the temperature range 30–800°C.

XPS (aka ESCA) measurements were performed in a ultra-high vacuum (UHV) multipurpose surface analysis system (SpecsTM model, Germany) operating at pressures <10^−10^ mbar using a conventional X-Ray source (XR-50, Specs, Mg-Kα, 1253.6 eV) in a “stop-and-go” mode to reduce potential damage due to sample irradiation. The survey and detailed Fe and Cu high-resolution spectra (pass energy 25 and 10 eV, step size 1 and 0.1 eV, respectively) were recorded at room temperature with a Phoibos 150-MCD energy analyzer. Powdered samples were deposited on a sample holder using double- sided adhesive tape and subsequently evacuated under vacuum (<10^−6^ Torr) overnight. Eventually, the sample holder containing the degassed sample was transferred to the analysis chamber for XPS studies. Binding energies were referenced to the C1s line at 284.6 eV from adventitious carbon. The curve deconvolution of the obtained XPS spectra were obtained using the Casa XPS program.

### Esterification activity assay

Valeric acid and ethanol were used as reaction substrates. In a typical esterification reaction, 30 mg of biosilicified lipase (15% Lipase m/v) was weighted and added to a solution of the alcohol (0.58, 1.16, 1.75 mL EtOH; 0.76, 1.53, 2.29 mL i-PrOH; 0.92, 1.83, 2.75 mL BuOH; corresponding to 1:1, 1:2, and 1:3 molar ratios respective to valeric acid), followed by the addition of 0.01 mol valeric acid (1.09 mL). The mixture was submerged in an ultrasonic bath (Ultrasound Midi II, P-seddlecta) and ultrasonicated for 2 h, with continuous temperature measurement. Reaction runs using the free enzyme were performed under identical conditions using comparable enzyme quantities (15% Lipase m/v), corresponding to 83.3 μL of lipase buffer solution (CAL-B, Sigma-Aldrich. Products were analyzed at different time intervals by GC in an Agilent 7890 GC model fitted with a capillary column Petrocol 100 m × 0.25 nm × 0.5 μm and a flame ionization detector (FID). The GC program was as follows: oven from RT to 220°C (40 min), inlet 250°C (split mode), Detector 300°C, N_2_ carrier flow: 30 mL min^−1^.

## Author contributions

SC-G conducted all experiments in the lab and wrote the first draft of the manuscript. AB and RL planned and programmed all experiments and wrote the final manuscript including the discussion.

### Conflict of interest statement

The authors declare that the research was conducted in the absence of any commercial or financial relationships that could be construed as a potential conflict of interest.
